# The manual mycobacteria growth indicator tube and the nitrate reductase assay for the rapid detection of rifampicin resistance of *M. Tuberculosis* in low resource settings

**DOI:** 10.1186/1471-2334-12-326

**Published:** 2012-11-27

**Authors:** Chamila P Adikaram, Jennifer Perera, Sandya S Wijesundera

**Affiliations:** 1 Department of Microbiology, Faculty of Medicine, University of Colombo, Kynsey Road, Colombo, 08, Sri Lanka; 2 Department of Biochemistry and Molecular Biology, Faculty of Medicine, University of Colombo, Kynsey Road, Colombo, 08, Sri Lanka

**Keywords:** Drug resistant tuberculosis, Determination of drug sensitivity, Manual MGIT, NRA in liquid medium, Agar proportion method

## Abstract

**Background:**

Tuberculosis (TB) is a disease of poverty that contributes significantly to ill-health in developing countries. Drug resistant TB is a major challenge to disease control. Early diagnosis and rapid determination of drug sensitivity is of paramount importance in eradication of TB. Although automated liquid culture based methods are available for rapid detection of drug resistance, the high cost of these tests prevent them from being used routinely in low resource settings. This study compares two phenotypic methods, the manual Mycobacteria Growth Indicator Tube (MGIT) and the Nitrate Reductase Assay (NRA) in liquid medium, with the agar proportion method (APM), the gold standard for susceptibility testing of *Mycobacterium tuberculosis*.

**Methodology:**

Fourteen day old *M. tuberculosis* strains (n=373) grown on solid media were used for drug susceptibility testing by APM, NRA and the manual MGIT method. Rifampicin free and rifampicin incorporated (final concentration, 1 μg/ml) media were inoculated with the recommended concentrations of mycobacterial suspensions and incubated at 37°C in 5% CO_2._ In the APM, the proportion of colonies in the drug containing medium was determined. In the NRA, the colour change in the medium was compared with a standard colour series after day 6 and day 12 of incubation. Growth in the MGIT was detected using the manual MGIT reader from day 2 onwards. The 2 methods were compared with the gold standard, APM to determine sensitivity and specificity and agreement between the methods was calculated using kappa statistics.

**Results:**

Thirty one (31) rifampicin resistant isolates were identified. When compared with the APM, the sensitivity of detection of rifampicin resistance was 85% for the NRA and 93% for the manual MGIT and the specificity was 99% and 100% respectively. Both assays, NRA (κ=0.86) and manual MGIT method (κ= 0.94) were in excellent agreement with the APM. The mean turnaround time for manual MGIT method and NRA were 08 days and 10 days respectively.

**Conclusion:**

The NRA in liquid medium and manual MGIT are useful alternatives to APM for drug susceptibility testing of *M. tuberculosis* in low resource settings.

## Background

The population of Sri Lanka is about 20 million and it is considered a low TB prevalence country in the Asian region. In Sri Lanka, the estimated incidence of all forms of tuberculosis in 2009 was 66 per 100,000 population. In 2009, 9643 new TB cases were notified and 5186 among them being sputum smear positive TB cases. The notification rate of TB was slightly increased when compared to the year 2008 [1]. The drug resistant rate in Sri Lanka is also low. It is around 0.2% among new TB patients and 18%–21% among re-treatment cases. The HIV co-infection rate among TB patients is currently estimated to be less than 0.1% [2].

Tuberculosis control activities in Sri Lanka operate through the National Programme for Tuberculosis Control & Chest Diseases (NPTCCD) which is a decentralized unit under the Ministry of Health. There are 26 chest clinics in the 25 administrative districts of Sri Lanka and the internationally recommended Directly Observed Treatment Short course (DOTS) strategy is used for treatment of TB patients throughout the island [1].

Multi Drug Resistant Tuberculosis (MDR-TB) is defined as resistance to isoniazid and rifampicin, the two most effective drugs among the currently used anti TB drugs [3]. Rifampicin is the important drug especially in the short-course treatment regimen. Significantly, rifampicin resistant isolates are also resistant to isoniazid, making rifampicin resistance a useful marker of MDR-TB [4,5]. Early and accurate diagnosis of MDR TB is very important for prevention and control of the disease. Currently several rapid and automated liquid culture methods such as the BACTEC 460 radiometric system and the MGIT 960 system for diagnosis of MDR-TB [6,7] have been commercialized [3]. However, these methods are beyond the reach of laboratories in most developing countries including Sri Lanka, due to high cost and the need for complex infrastructure facilities [8-11].

In Sri Lanka, drug susceptibility testing (DST) is carried out using the conventional proportion method on Lowenstein-Jensen (L-J) medium which requires a minimum of 28 days. This significantly delays the detection of drug resistance and appropriate treatment. Establishing a rapid culture based method for identification of rifampicin resistance is essential for control and prevention of MDR-TB. Therefore the objective of this study was to evaluate the suitability of the manual Mycobacteria Growth Indicator Tube and the nitrate reductase assay for the rapid detection of rifampicin resistance in a low resource setting.

## Methods

### *M. tuberculosis* strains

Three hundred and seventy three (373) isolates of *M. tuberculosis* cultured from suspected TB patients during the period March 2008 to September 2010 were used for the study. The reference strain, *M. tuberculosis* H37Rv and a known rifampicin resistant isolate confirmed by the National Tuberculosis Institute, Bangalore were used as quality control strains.

### Preparation of rifampicin solution

Rifampicin stock solution (10 mg/ml) was prepared by dissolving 10 mg of rifampicin powder (Sigma, USA) in 1 ml dimethyl sulphoxide (Sigma, USA). Filter sterilized aliquots of stock rifampicin solutions were kept at −70 °C until use. A working solution (1mg/ml) was prepared by diluting the stock solution with sterile double distilled water and used only once [12].

### Isolation of *M. tuberculosis* from clinical specimens

Sputum specimens were processed using the modified Petroff’s method and concentrated by centrifugation at 3500 g in a refrigerated (4°C) centrifuge for 15 minutes [13]. Sediment was diluted in 1 ml sterile distilled water. A small portion of the suspension was stained with Ziehl-Neelsen (ZN) stain and examined microscopically for the detection of acid fast bacilli [14]. Two slopes of L-J (Difco, US) (one containing paranitrobenzoic acid to detect *Mycobacterium* other than tuberculosis (MOTT) species) were inoculated with 100 μl of above bacterial suspension. The inoculated culture media were incubated at 37°C in 5% CO_2_ until growth was observed or discarded as negative after 8 weeks. Culture isolates were confirmed as *M. tuberculosis* if they were slow growing, non-pigment producing, reduced nitrate and did not grow in the presence of paranitrobenzoic acid. Further, species confirmation was carried out by PCR amplification of heat killed bacterial DNA [15] using primers derived from *IS* 6110 insertion element of *Mycobacterium* genome, pt18 (5'-GAA CCG TGA GGG CAT CGA GG-3') and INS2 (5'-GCG TAG GCG TCG GTG ACA AA-3') [16] (1^st^ base -Singapore).

### Drug susceptibility testing

#### Agar Proportion method

Agar proportion method (APM) remains the gold standard for culture based drug susceptibility testing for *M. tuberculosis*. The proportion method was carried out on Middlebrook 7H10 agar (Difco, US) plates as per CLSI guidelines [12]. Fourteen day old, fresh *M. tuberculosis* cultures grown on L-J medium were suspended in 7H9 broth medium to achieve a turbidity of McFarland No.1 standard. This suspension was further diluted (two fold and four fold) and used for inoculating rifampicin incorporated (1 μg/ml) agar plates and rifampicin free control agar plates. Colonies on each plate were counted on the 28^th^ and 42^nd^ day of inoculation, and the proportion of organisms growing in the presence of rifampicin was calculated using the following equation [12].

(1) $$\frac{\text{No}.\;\text{of colonies on drug containing medium}}{\text{No}.\;\text{of colonies on drug fee control medium}}\text{×}\;100$$

(Strains that showed ≥ 1% were considered as resistance to rifampicin)

#### Manual Mycobacterium Growth Indicator Tube (MGIT)

The MGIT (BD diagnostics, US) contains 4 ml of modified Middlebrook 7H9 broth with a fluorescence-quenching-based oxygen sensor embedded on the bottom of the tube. The level of fluorescence that the tube emits corresponds to the amount of oxygen consumed by organisms, which in turn is proportional to the number of bacteria present [17,18]. The rifampicin containing tubes (final rifampicin concentration of 1 μg/ml) were inoculated with 500 μl of a 1:5 dilution of a McFarland No: 0.5 bacterial suspension and rifampicin free control tubes were inoculated with 500 μl of a 1:500 dilution of McFarland No: 0.5 bacterial suspension as per manufacturer’s guidelines. The emission of fluorescence was measured using the manual MGIT reader from day 2 onwards. If the drug free control tube gave positive reading and the drug containing tube did not show a positive reading up to 15 days of inoculation the test strain was read as sensitive to rifampicin. The test was repeated when the drug free control tube failed to give a reading in the positive range within 13 days of inoculation. The presence of *Mycobacterium* in the test and control tubes was confirmed microscopically by ZN stain.

#### Nitrate Reductase Assay

**Figure 1:**
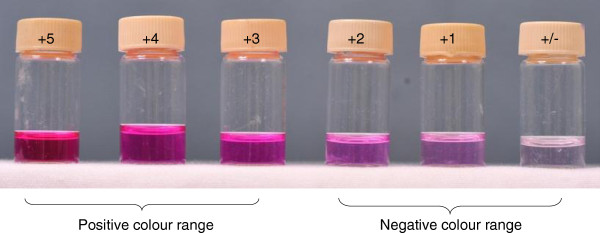
**Standard colour series for nitrate reductase assay (WHO, laboratory services in tuberculosis control culture part iii).** Colour range from +5 to +3 was considered as positive.

**Figure 2:**
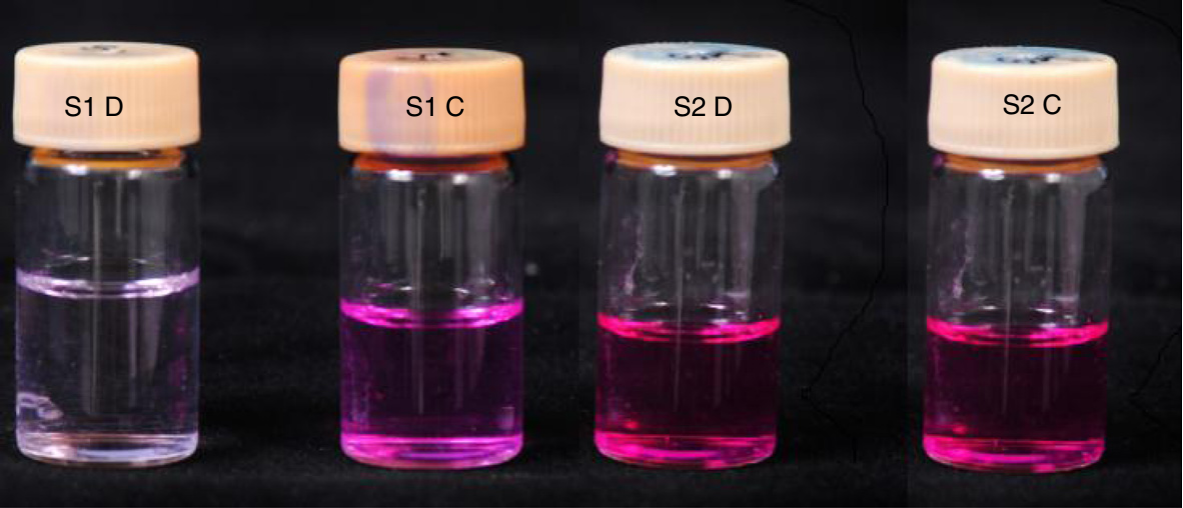
**An example of a test result of an isolate tested using NRA.** C-Rifampicin free control, D-Rifampicin containing medium, S1- Rifampicin susceptible strain of *M. tuberculosis*, S2- Rifampicin resistant strain of *M. tuberculosis.*

The nitrate reductase assay is based on the ability of *M. tuberculosis* to reduce nitrate to nitrite by the nitrate reductase enzyme and in the present study, NRA in liquid medium was evaluated as a drug susceptibility test method [19]. Middlebrook 7H9 broth medium with 0.1% sodium nitrate was used as the medium. The final concentration of rifampicin in the medium was 1 μg/ml. Each drug containing medium was inoculated with 100 μl of McFarland No: 1 bacterial suspension prepared from 14 day old *M. tuberculosis* strains grown on L-J medium. The control medium was inoculated with 100 μl of a 1:10 dilution of the same bacterial suspension [20]. Two sets of the drug free and drug containing media were inoculated per isolate. After 6 days, the first set was examined for a colour change by sequentially adding of Griess reagent (10 μl of 50% HCI, 20 μl of 0.2% sulfanilamide and 20 μl of 0.1% N-napthtylethylene-diamine) to the culture medium. Readings were obtained visually by comparing with the prescribed colour standards (Figure 1) [14]. Readings between +5 to +3 of the standard colour series (Figure 1) were considered positive. The drug free control medium was tested after 6 days and if it was negative, the second set was tested at day 12. If the drug free control gave a colour change within the positive range of the standard color series and the drug containing medium did not yield a colour change even after 12 days of incubation the isolate was considered sensitive to rifampicin (Figure 2). In order to prevent false negative results (if the produced nitrite have further reduced to nitric oxide), a small amount of powdered zinc (Sigma, USA) was added to each negative tube [21] and tested for generated nitrite in the medium by sequentially adding of HCI, sulfanilamide and N-napthtylethylene-diamine as described above. Development of a dark pink colour indicated the absence of the *M. tuberculosis* in the test medium. Bacterial contamination may cause false positive results. Therefore, to ensure that the media were free of contaminants the inoculated NRA broths were streaked on blood agar plates to detect any non-specific bacterial growth before testing for colour changing.

### DNA sequencing

The *rpoB* gene mutations, of the rifampicin resistant isolates, detected by any one of the phenotypic methods, were identified by DNA sequencing (Macrogen –Korea).

#### Data analysis

The suitability of the manual MGIT and the NRA in comparison with the APM was evaluated in terms of sensitivity (the ability to detect true drug resistance), specificity (the ability to detect true drug susceptibility), positive likelihood ratio and negative likelihood ratio. A positive likelihood ratio above 10 or a negative likelihood ratio below 0.1 was considered to indicate excellent test-performance, and ratios above 5 and below 0.2 were considered to indicate adequate performance. The agreement between the NRA results or the manual MGIT results, and the APM were estimated by kappa statistics. The kappa value(k), a measure of test reliability, was interpreted as follows: <0.2, poor; 0.21 to 0.4, fair; 0.41 to 0.6, moderate; 0.61 to 0.8, good; ≥0.81, excellent [22]. The consumable costs per test were calculated in determining the cost for each test method.

## Results

Thirty one rifampicin resistant isolates (resistant by one of the 3 methods) were identified among the 373 *M. tuberculosis* strains isolated during the study period March 2008 to September 2010. All 31 phenotypically resistant strains showed point mutations in the *rpoB* gene responsible for coding for rifampicin resistance (data not shown – see Additional file 1).

Twenty seven strains out of 31 were detected as rifampicin resistant by the APM, the currently used gold standard for conventional methods. MGIT and NRA identified 26 isolates each. There were 3 discordant results between the NRA and the APM and one discordant result between the MGIT and the APM. Three isolates that were detected as rifampicin resistant by NRA were susceptible by the manual MGIT and APM. Furthermore, one isolate that was rifampicin resistant by the manual MGIT was susceptible by APM and NRA (Table 1). Repeat testing of these 4 isolates provided the same results.

**Table 1:** Pattern of Individual strains (n=31) showing resistance to rifampicin with the DST methods used in the study

**Strain no (Lab No.)**	**DST method/s that confirmed rifampicin resistance**
C4, C6, C7, C8, C9, C10, C20, C73, C83, C86, C88, C115, M60, M127, M15, C27, C22 C23,C163, C254 C150, C135, C110	APM, MGIT and NRA
(n=23)	
M9, M33	APM and MGIT
(n=2)	
PCR 88,PCR 57	Only APM
(n=2)	
C120	Only MGIT
(n=1)	
M46 , C25, M22	Only NRA
(n=3)	

The sensitivity and specificity of the NRA in 7H9 broth medium when compared with the APM were 85% and 99% respectively (Table 2). Thus, there was a very good agreement between NRA and APM for detection of rifampicin resistance (κ= 0.86). An excellent agreement was also observed between the manual MGIT and APM (κ= 0.94) (Table 3) with 93% sensitivity and 100% specificity. The average turnaround time for MGIT and NRA in liquid medium was 08 days (mean) and 10 days (mean) respectively.

**Table 2:** Sensitivity, specificity, positive and negative likelihood ratio for NRA compared to APM (n=373)

**APM**	**NRA**		**Sensitivity %**	**Specificity %**	**Likelihood ratio**	
	**No.of resistant isolates**	**No.of susceptible isolates**			**Positive**	**negative**
Resistant (27)	23	4	85		98	0.15
Susceptible (346)	3	343		99		

**Table 3:** Sensitivity, specificity, positive and negative likelihood ratio for the MGIT method compared to APM (n=373)

**APM**	**MGIT**		**Sensitivity %**	**Specificity %**	**Likelihood ratio**	
	**No.of resistant isolates**	**No.of susceptible isolates**			**Positive**	**negative**
Resistant (27)	25	2	93		167	0.04
Susceptible (346)	1	345		100		

## Discussion

Rapid and accurate detection of drug resistance is a prerequisite for initiating effective anti-TB treatment. In Sri Lanka, presently, DST for *M. tuberculosis* is carried out only at the central reference laboratory, Welisara using solid based DST method. Liquid culture based or molecular based DST methods for detection of drug resistance are not yet available. Establishment of a more rapid DST method would positively impact the management of a patient harbouring a drug resistant strain. As rifampicin resistance is considered a surrogate maker of MDR TB, WHO recommends performing DST at least for rifampicin, especially in low resource settings [3].

In contrast to solid medium based DST methods, NRA and MGIT use an indicator to detect growth in the liquid medium, eliminating the need for visualization of growth as colonies. Therefore, NRA in liquid medium and MGIT methods are an attractive alternative to conventional methods. In this study, a good agreement was observed between APM and NRA in liquid medium or manual MGIT in the detection of rifampicin resistance. Similar results for detection of rifampicin resistance by manual MGIT and NRA have been reported previously [18,19,23,24].

The consumable cost per test for APM and NRA is around US$ 4.00 and US$ 3.00 respectively. Comparatively, the manual MGIT is more expensive (~US$ 7.00). However, both the NRA and the manual MGIT methods can be initiated with low technical expertise and initial cost. Additionally both methods are more rapid than the APM as the results of susceptibility testing will be available in less than 2 weeks.

In the evaluation of the manual MGIT for identification of rifampicin resistance, an in-house preparation of rifampicin solution was used instead of the commercially available rifampicin drug preparation kit (BD diagnostics, US). The appropriate volume of rifampicin solution was added to obtain a final concentration of 1 μg/ml of drug in the 4 ml broth medium. The excellent agreement between the MGIT and APM detecting rifampicin resistance confirms the suitability of using in-house preparation of drugs instead of commercially available drug kits that increases the cost of the test. The manual MGIT reader is a reliable and suitable instrument for use in low resource countries as no housing is required and the results can be read by placing the tube in the reading slot (See additional file 2). The time spent to take a reading is about 30 seconds. The cost of a MGIT reader is around US$ 3000 and it is a once only investment. Alternatively, in the absence of a manual MGIT reader a simple ultra violet (UV) lamp (365 nm) may be used to detect growth [25].

In the NRA, a standard colour series [14] was used to interpret test results. In the case of intermediate results, the test should be repeated for accurate interpretation. In our series, 2 of the 373 tests required repeat testing. The intermediate results in NRA may occur due to low inoculum in the medium. Contamination of the test medium can also lead to erroneous results as several other bacteria can reduce nitrate to nitrite. Therefore, it is important to ensure that there is no bacterial contamination, prior to reporting results of the NRA. Performing a purity test by sub culturing a blood agar plate with a loopful of test medium will prevent reporting of false positive test results due to contaminating bacterial flora.

## Conclusion

In conclusion, both the NRA in liquid medium and the manual MGIT agreed well with the APM in determination of rifampicin resistance. Introduction of these methods for low resource settings will make the determination of rifampicin resistance faster and cost effective. As the need for sophisticated instruments and high technical skills is minimal, the initial test establishment cost will be low. Therefore, the NRA in liquid medium and the manual MGIT are suitable alternatives to APM that can be used to determine rifampicin resistance especially in low resource settings.

### Ethics approval

The Ethics Review Committee of the Faculty of Medicine, University of Colombo, Sri Lanka approved the study (ERC Number is EC/06/062). The samples collected for the purpose of this research were from patients attending the chest clinic for diagnosis and treatment of tuberculosis and their informed consent was obtained prior to sample collection.

## Competing interests

The authors declare that they have no competing interests.

## Authors' contributions

CPA, AJP and WSSW designed the study; CPA carried out the laboratory work and analyzed the data; CPA, AJP and WSSW interpreted the data. CPA drafted the manuscript. AJP and WSSW supervised the work of CPA and revised the manuscript. All authors read and approved the final manuscript.

## Pre-publication history

The pre-publication history for this paper can be accessed here:

http://www.biomedcentral.com/1471-2334/12/326/prepub

## Supplementary Material

Additional file 1 **Phenotypic method/s detecting rifampicin resistance and the distribution of *rpoB *gene mutations of 31 isolates.** Click here for file

Additional file 2 Manual MGIT reader. Click here for file
