# Supplementary material for: The manual mycobacteria growth indicator tube and the nitrate reductase assay for the rapid detection of rifampicin resistance of M. Tuberculosis in low resource settings
Source: BMC Infect Dis. 2012 Nov 27;12:326. doi: 10.1186/1471-2334-12-326 (PMC3538674; doi:10.1186/1471-2334-12-326)
Supplement: Additional file 1 — Phenotypic method/s detecting rifampicin resistance and the distribution of rpoB gene mutations of 31 isolates. [file 1471-2334-12-326-S1.doc]

| Lab serial no. | ABST method that confirmed rifampicin resistant | codon showing *rpoB* gene mutation |
| --- | --- | --- |
| | C4 | | --- | | C6 | | C7 | | C8 | | C9 | | C10 | | C20 | | C73 | | C83 | | C86 | | C88 | | C115 | | M22 | | M60 | | M127 | | M46 | | M15 | | M9 | | PCR 88 | | PCR 57 | | C27 | | C22 | | C23 | | C25 | | C163 | | C254 | | M33 | | C150 | | C135 | | C110 | | C120 | | | APM+MGT+NRA | | --- | | APM+MGT+NRA | | APM+MGT+NRA | | APM+MGT+NRA | | APM+MGT+NRA | | APM+MGT+NRA | | APM+MGT+NRA | | APM+MGT+NRA | | APM+MGT+NRA | | APM+MGT+NRA | | APM+MGT+NRA | | APM+MGT+NRA | | NRA | | APM+MGT+NRA | | APM+MGT+NRA | | NRA | | APM+MGT+NRA | | APM+MGT | | APM | | APM | | APM+MGT+NRA | | APM+MGT+NRA | | APM+MGT+NRA | | NRA | | APM+MGT+NRA | | APM+MGT+NRA | | APM+MGT | | APM+MGT+NRA | | APM+MGT+NRA | | APM+MGT+NRA | | MGIT | | | 526 | | --- | | 526 | | 626 | | 626 | | 526 | | 526 | | 526 | | 526 | | 626 | | 526 and 626 | | 26 | | 526 | | 531 | | 531 | | 526 | | 526 | | 531 | | 626 | | 626 and 184 | | 626 | | 626 | | 526 | | 526 | | 526 | | 626 | | 626 | | 184 | | 626 | | 626 | | 526 | | 626 | |
