# Supplementary figures and images for: The manual mycobacteria growth indicator tube and the nitrate reductase assay for the rapid detection of rifampicin resistance of M. Tuberculosis in low resource settings
Source: BMC Infect Dis. 2012 Nov 27;12:326. doi: 10.1186/1471-2334-12-326 (PMC3538674; doi:10.1186/1471-2334-12-326)

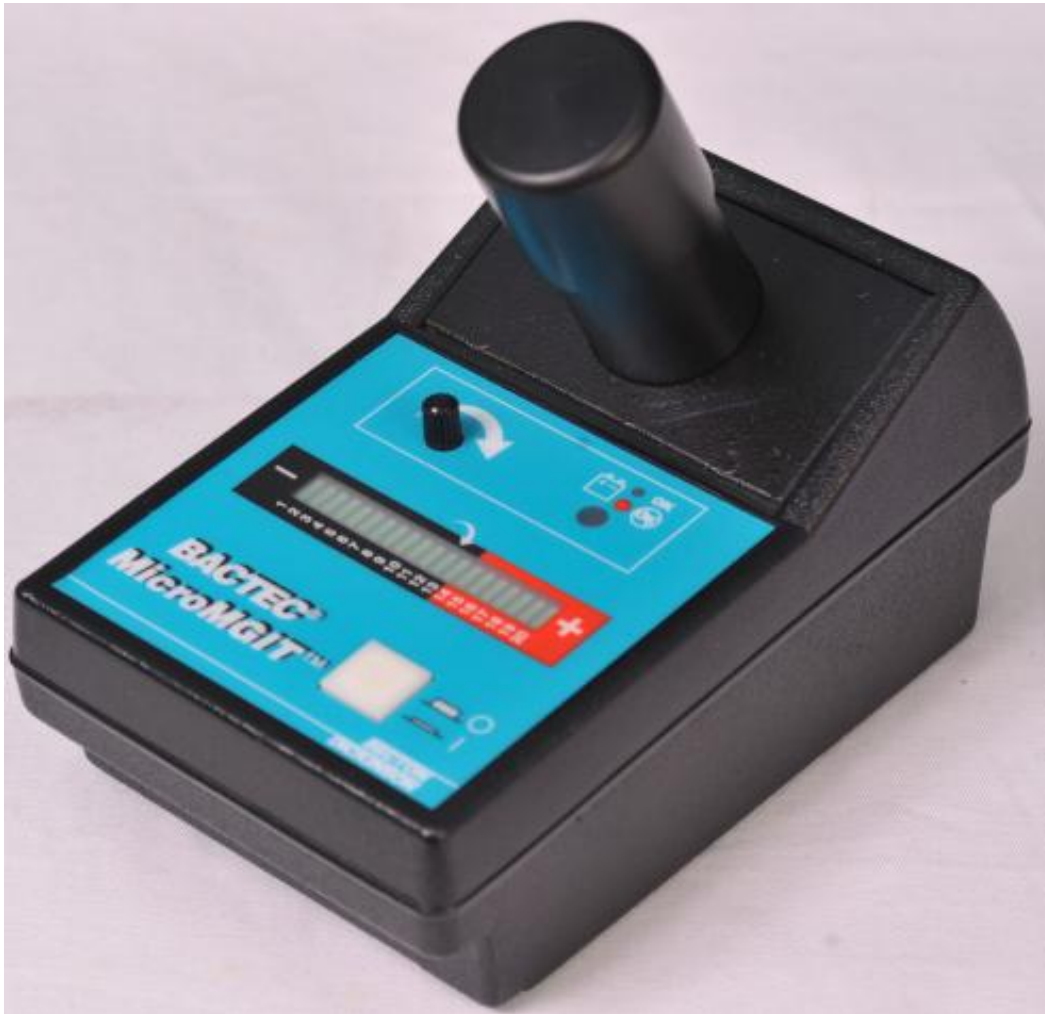

Supplement: Additional file 2 — Manual MGIT reader. [file 1471-2334-12-326-S2.pdf]
